# Revisiting the role of CD4^+^ T cells in cancer immunotherapy—new insights into old paradigms

**DOI:** 10.1038/s41417-020-0183-x

**Published:** 2020-05-27

**Authors:** Rong En Tay, Emma K. Richardson, Han Chong Toh

**Affiliations:** 1 Singapore Immunology Network, Agency for Science, Technology, and Research (A*STAR), Singapore, 138648 Singapore; 2 Division of Medical Oncology, National Cancer Centre Singapore, Singapore, 169610 Singapore

**Keywords:** Tumour immunology, Cancer immunotherapy, Tumour immunology

## Abstract

Cancer immunotherapy has revolutionised cancer treatment, with immune checkpoint blockade (ICB) therapy and adoptive cell therapy (ACT) increasingly becoming standard of care across a growing number of cancer indications. While the majority of cancer immunotherapies focus on harnessing the anti-tumour CD8^+^ cytotoxic T cell response, the potential role of CD4^+^ ‘helper’ T cells has largely remained in the background. In this review, we give an overview of the multifaceted role of CD4^+^ T cells in the anti-tumour immune response, with an emphasis on recent evidence that CD4^+^ T cells play a bigger role than previously thought. We illustrate their direct anti-tumour potency and their role in directing a sustained immune response against tumours. We further highlight the emerging observation that CD4^+^ T cell responses against tumours tend to be against self-derived epitopes. These recent trends raise vital questions and considerations that will profoundly affect the rational design of immunotherapies to leverage on the full potential of the immune system against cancer.

## Introduction

Cancer immunotherapy has advanced rapidly in the clinic in recent years because of two main therapeutic drivers: immune checkpoint blockade (ICB) therapy using antibodies blocking inhibitory receptors of the immune system across tumours [1–4], and adoptive cell therapy (ACT) using T cells engineered to express chimaeric antigen receptors (CAR T cells) targeting blood malignancies [5, 6]. These therapeutic modalities have largely focused on boosting the quantity and quality of anti-tumour CD8^+^ cytotoxic T lymphocyte (CTL) responses to generate therapeutic benefits. However, despite ongoing efforts to extend the therapeutic reach and increase the safety of ICBs and ACT [3, 4, 7–9], typically by investigating the therapeutic potential of rationally designed combination therapies (e.g. tumour vaccines with ICBs, radio- and chemotherapy with ICBs etc.) [10–13], there remain significant limitations in the clinical efficacies of both these treatment modalities.

Recently, it has become increasingly clear that CD4^+^ T cells play a critical role in developing and sustaining effective anti-tumour immunity, even in cancer immunotherapies specifically designed to activate a CD8^+^ CTL response. In this review, we discuss new developments detailing the multifaceted involvement of CD4^+^ T cells in the anti-tumour immune response and revisit older paradigms on the roles of CD4^+^ T cells in tumour immunity. Finally, we will highlight some novel emergent aspects of anti-tumour CD4^+^ T cells and offer our perspective on future directions to accelerate translation of this knowledge into clinical therapies.

## A brief history of trends in cancer immunotherapy targeting CD4^+^ T cells

**Fig. 1:**
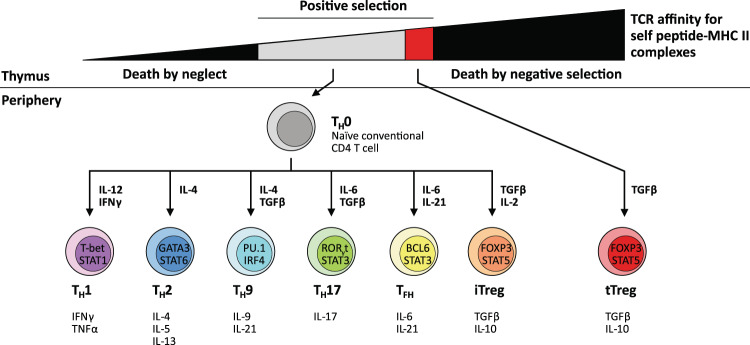
Development of CD4 T cells and functional diversity of CD4 subsets in immunity. CD4^+^ T cells are T lymphocytes that express T cell receptors (TCRs) recognising peptide antigens presented in the context of Class II major histocompatibility complex (MHC II) molecules. CD4^+^ T cells express the TCR co-receptor CD4, which binds to the β2 domain of MHC II and facilitates TCR engagement with peptide-MHC II complexes on antigen-presenting cells [111]. During thymic development, the cell fate of developing thymocytes is decided by their TCR affinity for self-peptide-MHC complexes presented by thymic epithelial cells. Thymocytes that have little to no affinity for self-peptide do not initiate activating signals from their TCR complexes and thus die by neglect. Conversely, thymocytes with high self-reactivity are negatively selected and deleted by apoptosis. Thymocytes with intermediate TCR affinities below the negative selection threshold receive positive selection via activating TCR signals and complete thymic maturation as naïve conventional T cells (T_H_0). Some thymocytes with moderately high affinities to self-antigen are redirected into the regulatory T cell (Treg) developmental pathway, where they acquire immunosuppressive function to regulate tissue homoeostasis and resolution of immune responses [53, 112]. Upon receiving cues from the cytokine milieu together with TCR activation, naive CD4^+^ T cells upregulate expression of key transcription factors regulating subset differentiation, which in turn drive the expression of major effector cytokines associated with each particular subtype [113, 114]. Key transcription factors and cytokines involved are indicated for individual subtypes. CD4^+^ T cells augment the development of the CTL response [21, 24] and are required for the development of CD8^+^ T cell immunity (reviewed extensively here [115]) in their role as central co-ordinators of adaptive immunity. Unlike CD8^+^ T cells, whose primary function is to mediate cell contact-dependent cytotoxicity of infected or malignant cells, CD4^+^ T cells exhibit a diverse repertoire of effector functions and exhibit considerable phenotypic plasticity and heterogeneity depending on local context and microenvironment [113, 114]. CD4^+^ T cells activated in the periphery can also differentiate into induced Tregs (iTregs), which are able to mediate immunosuppression similar to thymic Tregs (tTregs).

**Fig. 2:**
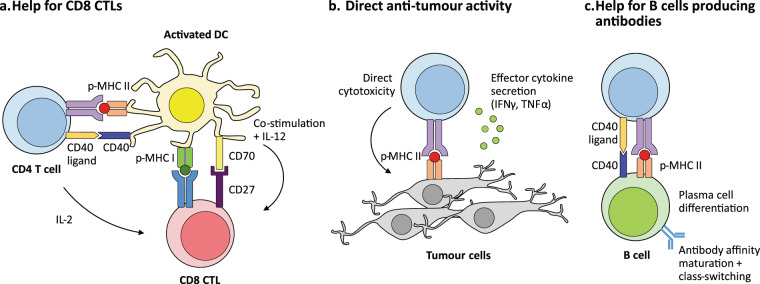
Multifaceted roles of CD4^+^ T cells in anti-tumour immunity. CD4^+^ T cells play key roles in tumour immunity through several different mechanisms. **a** A major role of CD4^+^ T cells is the provision of help for anti-tumour CTLs through both direct and indirect mechanisms (discussed in-depth here [116]). Activated CD4^+^ T cells secrete interleukin (IL)-2, which directly activates CD8^+^ CTLs expressing the high-affinity IL-2 receptor α subunit (CD25) by driving their effector function, differentiation, and proliferation. CD4^+^ T cells also indirectly provide help for the anti-tumour CD8^+^ CTL response by supporting and maintaining pro-inflammatory cross-presenting dendritic cells (DCs) [101], which in turn provide the three activating signals for CD8^+^ CTLs [115, 117]. This is primarily mediated by the upregulation of CD40 ligand (CD154) [22, 23, 118] on activated CD4^+^ T cells, which engages its cognate receptor CD40 on DCs to induce and maintain the type I profile of DCs (expression of B7 family ligands, CD70, and secretion of IL-12) [116]. These signals strongly induce anti-tumour effector functions in CD8^+^ CTLs such as the acquisition of cytotoxicity and the secretion of tumoricidal cytokines such as interferon-γ (IFNγ), and also stimulate the effector [117, 119, 120] and memory [121–124] phenotype differentiation of CD8^+^ T cells. **b** CD4^+^ T cells also produce effector cytokines such as IFNγ and tumour necrosis factor-α (TNFα), which have direct anti-tumour activity, following activation and polarisation into the T_H_1 phenotype [125] in response to signals from DCs, particularly IL-12. In addition, CD4^+^ T cells can mediate direct cytotoxicity against tumour cells in a similar manner to their CD8^+^ T cell counterparts under specific conditions in both preclinical mouse tumour models [45, 46, 126] and in patient-derived CD4^+^ T cells [127]. **c** CD4^+^ T cells are also indispensable for the induction of humoral responses against tumour antigens by providing help via CD40 ligand signalling to CD40 on B cells to drive their differentiation and maturation into affinity-matured, class-switched plasma cells. Their activity correlates with the presence of serum antibodies specific to tumour antigens [17, 128], and they likely play a role in driving local antibody responses in tertiary lymphoid structures [129] adjacent to solid tumours.

CD4^+^ T cells are highly versatile, polyfunctional cells that constitute the second arm of adaptive T cell immunity alongside their sister lineage of CD8^+^ cytotoxic T cells. CD4^+^ T cells can differentiate into one of several diverse functional subtypes in response to context-dependent signals (Fig. 1), which in turn allows them to provide ‘help’ to appropriate effector immune cells in their primary role as central co-ordinators of the immune response. CD4^+^ T cells primarily mediate anti-tumour immunity by providing help for CD8^+^ CTL and antibody responses, as well as via secretion of effector cytokines such as interferon-γ (IFNγ) and tumour necrosis factor-α (TNFα), and, under specific contexts, via direct cytotoxicity against tumour cells (Fig. 2). The earliest efforts to induce CD4^+^ T cell responses against tumours were attempts to generate T_H_1-polarised CD4^+^ T cells by vaccination with peptide epitopes (Table 1). These peptides were typically derived from highly immunogenic tumour-associated antigens [14, 15] including members of the cancer testis antigen family such as NY-ESO1 [16, 17] or melanoma-associated antigens such as MAGE-A3 [18]. In particular, these studies focused on boosting CD4^+^ T cell-derived secretion of T_H_1-characteristic tumoricidal cytokines (e.g. IFNγ) as a readout of increased anti-tumour CD4^+^ responses. Other variations of this strategy were attempts to isolate and expand tumour-reactive CD4^+^ T cells from patient tumour-infiltrating lymphocytes (TILs) using tumour-derived antigens with major histocompatibility complex (MHC) II-restricted epitopes and then re-infusing them as a form of ACT [19, 20], or more recently, by engineering autologous CD4^+^ T cells from cancer patients to express synthetic chimaeric antigen receptors that recognise antigenic epitopes on tumour cells.

**Table 1:** Examples of tumour-associated antigens with MHC II-restricted epitopes cited in this review.

Tumour-associated antigen(s) with MHC II-restricted epitopes	Species	References cited in this study
*MUC1*, Mucin 1	Human	[14, 28]
Cancer embryonic antigen (CEA) family	Human	[15, 28]
NY-ESO1 cancer testis antigen	Human	[16, 17]
Melanoma antigen gene-A (MAGE-A) family	Human	[18, 28]
*TYR*, Tyrosinase	Human	[29]
*PMEL*, Premelanosome protein (gp100)	Mouse [26], Human [29]	[26, 29]
*ERBB2IP*, Erbb2 interacting protein	Human	[20]
*HER2/neu*, Human epidermal growth factor receptor 2	Human	[28, 30]
*BIRC5*, Survivin	Human	[28, 31–33]
*TERT*, Telomerase reverse transcriptase	Human	[28, 34–36]

In contrast, although the development and effector functions of distinct subsets of CD4^+^ T cells has been recognised and described (Fig. 1), the interplay between polyfunctional CD4^+^ T cells and other immune cell lineages within the context of tumour immunity is less well understood. This is despite the fact that the critical role for CD4^+^ T cells in supporting the effector function and differentiation of CD8^+^ T cells had been described as early as the 1980s [21] and was already well-established by the late 1990s [22–25]. The first major turning point began in the mid-2000s when mouse models of cancer demonstrated that CD4^+^ T cells were necessary to maintain and sustain anti-tumour CD8^+^ CTL responses [26, 27] (Fig. 2). This is also validated by observations from clinical studies, most commonly in the context of cancer vaccines [28–30].

Parallel to this revival of interest in CD4^+^ T cells, there has also been significant research directed at producing a universal cancer vaccine based on promiscuous class II epitopes from self-molecules such as survivin [31–33] (an inhibitor of apoptosis) and telomerase reverse transcriptase (TERT) [34–36] (Table 1). Even at this rudimentary stage, it was already recognised that spontaneous CD4^+^ T cell responses towards self-antigens could be harnessed to boost anti-tumour immunity.

## (Re)discovery of the importance of CD4^+^ T cells in driving and sustaining anti-tumour immune response

Despite these encouraging early findings, the fundamental role of CD4^+^ T cells in orchestrating anti-tumour responses was until recently eclipsed by the clinical success of CD8^+^ T cell-based immunotherapies. This greater attention to CD8^+^ T cells was partly due to greater availability of tools such as tetramers that could be used to monitor CD8^+^ responses, and also partly because CD8^+^ T cell numbers and function were the most proximal readouts of anti-tumour immunity. However, the last 5 years have seen many reports recognising the critical role of CD4^+^ T cells in driving anti-tumour immunity and in supporting anti-tumour CD8^+^ T cell responses.

In 2015, Linnemann et al. found that human melanomas frequently contained mutant neoepitopes recognised by CD4^+^ cells [37]. This was quickly followed by a report from the lab of Özlem Türeci and Ugur Sahin, which demonstrated that immunogenic tumour mutations in the ‘mutanomes’ of three separate preclinical mouse tumour models largely induced a CD4^+^ T cell response [38], not a CD8^+^ T-cell response as had been expected. Two years later, the same group and Catherine Wu’s group published back-to-back reports reporting clinical findings showing that personalised neoantigen vaccines for melanoma patients primarily induced tumour-specific responses in CD4^+^ rather than CD8^+^ T cells [39, 40]. In both cases, synthetic long peptides (SLPs) were used as the mode of vaccination. These findings serendipitously validated findings from 10 years prior that immunisation with longer peptides induced a sustained CD8^+^ T cell response [41], likely due to CD4^+^ T cell help, whereas immunisation with exact-length MHC I-restricted peptides (specifically targeting only CD8^+^ T cells) only gave rise to a fleeting CD8^+^ T cell response. Ott et al. further theorised that the unexpected preponderance of CD4^+^ over CD8^+^ T cell responses could have been due to (1) a relative paucity of the cross-presenting dendritic cell subset within tumours, which led to more efficient priming of CD4^+^ relative to CD8^+^ T cells, and (2) the relatively higher promiscuity of MHC II-restricted epitopes due to more relaxed binding requirements compared with MHC I-restricted epitopes [39].

Concurrently, findings from preclinical mouse models also highlight a larger, more fundamental role for CD4^+^ T cells in anti-tumour immunity than was previously thought. In 2017, Spitzer et al. reported that a unique T_H_1-like CD4^+^ subset was expanded in non-tumour peripheral tissues during an active anti-tumour response to adjuvant therapy [42], in a study conducted in the Py-MMTV mouse model of spontaneous mammary tumours. They further demonstrated that this population of CD44^+^ CD69^+^ CD62L^−^ CD27^lo^ T-bet^+^ CD4^+^ T cells conferred a protective benefit when transferred into treatment-naive tumour hosts. In addition, this group also found an analogous population of CD4^+^ T cells in the peripheral blood of melanoma patients who had received ipilimumab (α-CTLA-4 blocking antibody) combined with granulocyte-macrophage colony-stimulating factor (GM-CSF) therapy.

In 2019, two separate preclinical studies independently highlighted the role of CD4^+^ T cells in enhancing the anti-tumour CD8^+^ T-cell response. Zander and colleagues identified a critical role for CD4^+^ T cell-derived interleukin (IL)-21 in driving the differentiation of a CX3CR1^+^ cytotoxic effector CD8^+^ T cell subtype with enhanced anti-viral and anti-tumour activity against murine B16F10 melanoma tumours (an immunologically-cold, aggressive tumour that is refractory to ICB therapy) [43]. Similarly, Alspach et al. found that poorly immunogenic tumours engineered to express MHC II-restricted antigens could induce T_H_1-polarised anti-tumour CD4^+^ T cell responses. These antigen-specific CD4^+^ T cells enhanced the efficacy of anti-tumour CD8^+^ T cell responses, and mediated long-lived protection against subsequent tumour re-challenge in mice that survived primary tumour challenge [44].

In addition, in a recent study, Śledzińska et al. built on previous work from a decade ago [45, 46] and found that tumour-infiltrating T_H_1-like CD4^+^ T cells acquired cytotoxicity against B16 melanoma. This development of cytotoxic capability required expression of the transcription factors T-bet and Blimp-1 [47]. Collectively, these recent preclinical studies demonstrate the critical and versatile role of polyfunctional tumour-infiltrating CD4^+^ T cells in the overall anti-tumour immune response.

Recent clinical evidence has also raised the importance of CD4^+^ T cells in generating successful anti-tumour immunity. In a meticulous deep single-cell analysis of T cell receptor (TCR)- and RNA-sequencing from colorectal cancer patient biopsies, Zemin Zhang’s group found that patients with microsatellite-instable tumours (which show a strikingly favourable response profile to ICB therapy) showed preferential enrichment for a T_H_1-like subset of CD4^+^ T cells. These unique tumour-infiltrating CD4^+^ T cells expressed the transcription factor *BHLHE40*, the effector cytokine *IFNG*, and the chemokine receptor *CXCR5* [48]. In a second study, Galaine et al. reported the presence of anti-tumour CD4^+^ T cells that recognised MHC II-restricted, promiscuously-binding tumour-associated antigens in colorectal cancer patients undergoing oxaliplatin chemotherapy. In some patients, CD4^+^ T cell responses persisted even after 3 months of oxaliplatin treatment [49], highlighting the importance of understanding the immunomodulatory effects of oxaliplatin and other chemotherapeutic agents on CD4^+^ T cells.

Interestingly, the presence of specific subsets of CD4^+^ T cells in the peripheral circulation was also found to be predictive of good prognosis in non-small cell lung cancer (NSCLC) patients, where ICB treatment has efficacy either as a single agent or in combination therapy. A Japanese study by Kagamu et al. found that a higher level of circulating CD62L^lo^ CD4^+^ T cells prior to PD-1 checkpoint blockade was significantly correlated with better response and with the presence of effector CD8^+^ T cells [50]. This subset of CD4^+^ T cells expressed T-bet and CXCR3 but not CD27 or FoxP3. Furthermore, the maintenance of high levels of these CD4^+^ T cells correlated significantly with patient survival, whereas a loss of this population of CD4^+^ T cells after ICB was correlated with resistance to ICB therapy. Separately, Laheurte et al. found that higher levels of TERT-specific T_H_1-type CD4^+^ T cells in peripheral blood was associated with better prognosis of NSCLC patients [51].

Overall, these recent clinical advances corroborate the robust findings from preclinical models that CD4^+^ T cells play a fundamental role in driving and sustaining meaningful anti-tumour immune responses.

## Regulatory T cells in cancer immunotherapy—a plot twist

CD4^+^ T regulatory cells (Tregs) are a major subset of CD4^+^ T cells, distinct from the conventional CD4^+^ effector lineage (Tconvs), that mediate immunosuppressive and tolerogenic functions in both homoeostasis and inflammation [52–56]. CD4^+^ Tregs are most broadly characterised by their expression of the transcription factor FoxP3, which is a master regulator of their immunosuppressive function [53] (Fig. 1). Until recently, the prevailing paradigm was that the presence of Tregs within the tumour microenvironment (TME) was ‘bad’ for anti-tumour immunity. Tregs suppress anti-tumour immune effector responses in the TME, primarily by promoting an immunosuppressive microenvironment by their secretion of cytokines such as IL-10 and transforming growth factor-β (TGFβ) [57–59], and possibly by targeting anti-tumour effector immune cells and antigen-presenting cells for granzyme- and perforin-mediated killing [59–61]. In addition, it has also been proposed that the milieu of the tumour microenvironment converts effector CD4^+^ T cells into Tregs or promotes the differentiation of naïve CD4^+^ T cells into induced Tregs [62, 63], further exacerbating suppression of nascent anti-tumour immunity. The immunosuppressive role for tumour-infiltrating Tregs continues to be validated by observations in the clinic that increased frequencies of Tregs are associated with poorer cancer patient prognoses [64–67].

Consequently, most therapeutic modalities targeting Tregs involve depletion by specific chemotherapeutic agents such as cyclophosphamide, or by antibody-dependent cellular cytotoxicity (ADCC) mechanisms initiated by the targeted labelling of Tregs with antibodies specific for surface markers strongly expressed on Tregs such as CD25 and CTLA-4 (comprehensively reviewed here [68]). Other approaches include blocking of Treg recruitment into the TME by blocking the binding of chemokine receptors such as CCR4 involved in their trafficking to tumour sites [68, 69], or inhibiting Treg immunosuppressive function [59, 68]. Of note, CD4^+^ Tregs constitutively express high levels of surface receptors that are only upregulated by conventional T cells in response to activation, including PD-1 and CTLA-4, as well as a host of TNF receptor superfamily members such as OX-40 (CD134) and GITR [57, 58, 70]. These receptors are potential targets for antibody-mediated depletion. Of note, the therapeutic efficacy of the anti-CTLA-4 antibody ipilimumab is likely due in part to its depleting effects on intratumoral Tregs [71].

However, despite advances in technology, the function and stability of Tregs within the tumour microenvironment have been poorly characterised beyond the minimum knowledge necessary to remove Tregs or inhibit their function.

Early reports as far back as 2009 indicated potential involvement of FoxP3^+^ CD4^+^ Tregs in the anti-tumour response in patients that had been treated with a MHC II-restricted MAGE-A3 peptide vaccine [18]. A subsequent study in murine B16F10 melanoma found that administration of glucocorticoid-induced TNF receptor (GITR) agonist antibodies resulted in the selective expansion of tumour-specific Tregs and was accompanied by a broadening of the TCR repertoire of the Teg population, but not of the Tconv TCR repertoire [72]. Furthermore, Tregs were substantially increased in HER2^+^ breast cancer patients that showed tumour rejection following treatment with the drug-antibody conjugate trastuzumab emtansine (Kadcyla®) [73]. Although tumour-infiltrating Tregs were known to be immunosuppressive, distinct from anti-tumour CD4^+^ Tconvs [67, 74, 75], these early studies raised the question of whether tumour antigen-specific Tregs could potentially acquire an effector phenotype under certain conditions and contribute towards anti-tumour immunity.

Recent literature has raised the interesting possibility that Tregs can indeed be converted into anti-tumour effector cells. Several molecular mechanisms controlling the stability of the suppressive phenotype of Tregs have been identified, including OX-40 signalling [76, 77], GITR signalling [78], NF-κB signalling [79], the histone methyltransferase Ezh2 [80], and the Ikaros family transcription factor Helios (*IKZF2*) [81, 82]. These molecules may well function as gatekeepers for the conversion of Tregs into anti-tumour effector CD4^+^ T cells. The application of such findings to develop Treg conversion as a potential cancer immunotherapy could thus potentially deliver a potent one-two combo by minimising Treg immunosuppression while simultaneously generating more anti-tumour effector T cells.

In one notable study, Shimon Sakaguchi’s group showed that CD4^+^ Tregs isolated from colorectal cancer patients could be subdivided into two functionally distinct populations based on levels of FoxP3 expression. These tumour-infiltrating Tregs consisted of a FoxP3^hi^ population suppression-competent population, and a second poorly suppressive FoxP3^lo^ population that was induced by the T_H_1-polarising cytokine IL-12 and that also secreted the pro-inflammatory cytokines IFNγ and IL-17 [65]. Interestingly, patients with higher infiltrates of FoxP3^lo^ cells showed better clinical prognoses than did patients with lower infiltrates of the same cells, suggesting that these cells could be anti-tumour effectors. More recently, Steven Rosenberg’s group found that tumour-infiltrating CD4^+^ Tregs expressed a distinct TCR repertoire that was enriched in tumours across several cancer types [83] by using deep TCR-sequencing to compare the TCR repertoires of tumour-infiltrating CD4^+^ Tregs and Tconvs with those of CD4^+^ T cell populations in autologous peripheral blood. Intriguingly, the authors also found two patient-derived TCRs that were reactive to tumour neoantigens and stimulated production of the effector cytokine IFNγ. These findings, while not definitive statements about tumour-reactive Tregs, provide support for Treg conversion as a paradigm relook at cancer immunotherapy.

## Recent advances in CD4^+^ CAR T cells

The observation that CD4^+^ T cells synergise with CD8^+^ T cells in the immune response against tumours also extends to CAR T cell adoptive immunotherapy. Carl June’s research group recently reported preliminary observations that a higher CD4/CD8 ratio in the leukapheresis products used to generate CAR T cells directed against multiple myeloma correlated with better clinical response [84]. These observations, while not necessarily indicative of any particular mechanism, are nonetheless consistent with the known function of CD4^+^ T cells in co-ordinating and sustaining the immune response against tumours. Furthermore, in a recent glioblastoma (GBM) study, Wang et al. found that the maintenance of CD4^+^ CAR T cells correlated positively with the recursive killing ability of CAR T cell products derived from GBM patients (so-called “serial killers [8]”). These CD4^+^ CAR T cells also showed anti-tumour effector activity independent of CD8^+^ CAR T cells [85].

Reflecting the increasing appreciation for the role of CD4^+^ T cells in sustaining the efficacy of CAR T cell therapy, a number of recent studies have characterised molecular regulators of CD4^+^ CAR T function. In an elegant study, Yang et al. showed that CD4^+^ and CD8^+^ T cells transduced with a second-generation CD19-targeting CAR (with a CD28 co-stimulatory signalling domain) showed similar in vitro and in vivo efficacy against a murine model of pre-B cell acute lymphoblastic leukaemia. Strikingly, unlike CD8^+^ CAR T cells that become exhausted or apoptotic when exposed to activating signals through both their CARs and native TCRs, CD4^+^ CAR T cells retained their in vivo efficacy in controlling leukaemia [86]. In another study using murine CAR T cells specific for B7H6 (a tumour-specific activating ligand for the NKp30 receptor [87]), the T_H_1 phenotype-associated transcription factor T-bet was found to increase the efficacy of their CAR T cells in controlling NKp30^+^ RMA tumours in mice [88].

Finally, in a comprehensive study combining multiple single-cell analysis techniques, Xhangolli and co-authors profiled the transcriptional responses of human T cells expressing a third-generation CAR specific for CD19 (containing both 4-1BB and CD28 signalling domains) in response to CAR stimulation. In agreement with the work of Yang et al, 2 years prior, they found that CD4^+^ and CD8^+^ CAR T cells were indeed equally effective at killing human CD19^+^ Raji cells. Furthermore, while both CD4^+^ and CD8^+^ CAR T cells were highly polyfunctional, with more than 50% secreting >5 cytokines (including the T_H_1-characteristic cytokine IFNγ), CD4^+^ CAR T cells were slightly more polyfunctional than CD8^+^ CAR T cells, and exhibited a mixed T_H_1/T_H_2 transcriptional and cytokine secretion profile [89].

Altogether, these recent studies highlight a role for CD4^+^ T cell-derived CAR T cells distinct from their CD8^+^ T cell-derived counterparts. Although molecular evidence is currently scant and preliminary, these pioneer studies also suggest that a polyfunctional T_H_1-like phenotype (characterised by secretion of IFNγ and possibly driven by the activity of the transcription factor T-bet) may be beneficial for the overall efficacy of the final CAR T cell product. However, given the diversity of CD4^+^ T cell functional subtypes (Fig. 1) and the current lack of information regarding potential cross-talk between CAR signalling and CD4^+^ T cell-intrinsic gene programmes, it is very likely that CD4^+^ T cell-derived CAR T cells polarised towards non-T_H_1 phenotypes may also mediate effective anti-tumour immunity in a context-dependent manner. In addition, there is also the possibility that CD4^+^ CAR T cells could be influenced by the TME to acquire Treg-like immunosuppressive phenotypes. Further investigation into all these open questions would be essential to further refine CAR T cell engineering to increase its treatment efficacy and safety profile.

## Self-reactive anti-tumour CD4^+^ T cells—the next frontier in cancer immunotherapy?

The evolving themes we have highlighted illustrate a renewed appreciation of the central role of CD4^+^ T cells directed against cancer. While these recent studies have clearly established that CD4^+^ T cells provide critical help for anti-tumour immune responses, the repertoire of antigens that CD4^+^ T cells recognise within the tumour microenvironment remains relatively unexplored.

In principle, tumour-derived MHC II-restricted epitopes could be derived either from tumour-specific mutations or from self-antigens. The empirical evidence thus far suggests that the majority of the anti-tumour CD4^+^ response is directed against self-derived epitopes, regardless of whether Tconv or Treg cells respond. This is likely due to the fact that self-reactive CD4^+^ T cells are less likely to be deleted during formation of central tolerance in the thymus (as are self-reactive developing CD8^+^ T cells), but rather are tolerised in the periphery [55] or develop into regulatory T cells [53, 54, 56].

**Fig. 3:**
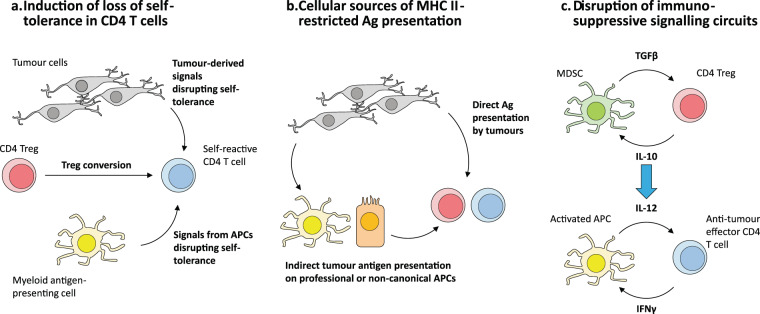
Open questions for research into harnessing the therapeutic potential of tumour-specific CD4^+^ T cells. **a** The majority of tumour-reactive CD4^+^ T cells have been found to recognise self-derived antigens, but have thus far only been shown to become activated in the tumour microenvironment (TME) and not in surrounding tissues, suggesting that there may be mechanisms specific to within the TME that permit the breaking of self-tolerance. Another possible avenue for loss of CD4^+^ T cell self-tolerance is the conversion of self-specific Tregs into conventional effector T cells within the TME. **b** Because CD4^+^ T cells are MHC II-restricted, their activation within the TME requires antigen presentation in the context of MHC II molecules. In principle, MHC II^+^ tumours could directly present antigen and activate CD4^+^ T cells, or antigen presentation could occur indirectly via antigen-presenting cells (APCs) resident within tumours or tumour-draining lymphoid sites. **c** The mutually reinforcing interaction between myeloid-derived suppressor cells (MDSCs) and regulatory CD4^+^ T cells (Tregs) (above) is a negative mirror image of the APC-effector CD4^+^ T cell synergy that drives the generation of effective immunity (below). Understanding the molecular circuitry is crucial to developing targeted strategies to disrupt and convert these negative interactions into cycles that drive anti-tumour immunity.

This raises three interesting questions that could potentially open up new approaches towards more effectively incorporating CD4^+^ T cells into the cancer immunotherapy arsenal (Fig. 3).

First, what breaks peripheral tolerance in self-reactive CD4^+^ T cells within the tumour microenvironment (Fig. 3a)? Many of the anti-tumour CD4^+^ T cell responses described in the early days of cancer immunotherapy were specific for highly immunogenic self-derived antigens [14, 31, 32, 90–93]. This in turn implies that, in the case of self-reactive anti-tumour CD4^+^ T cells, the self-tolerance mechanisms that would normally check such aberrant self-directed autoimmunity are either disrupted or negated within the TME. Recent evidence in mouse models has shown that Tregs (likely to be self-reactive) can become anti-tumour effector cells in response to epigenetic modulator drugs [80] or agonist antibodies specific for TNF receptor superfamily members [82]. Investigating the upstream signalling cues that trigger and maintain a pro-inflammatory CD4^+^ T cell phenotype could lead to the identification of therapeutic regimens that favour the generation and/or maintenance of self-antigen-biased, anti-tumour CD4^+^ T cell immunity.

Furthermore, we posit that elucidating the origin of self-antigen-directed CD4^+^ T cell immunity would also be particularly relevant to the engineering of more sophisticated CAR T cell products for cellular therapy. Certainly, it would open up the possibility of using self-antigen-specific CARs to redirect autologous CD4^+^ T cells (possibly even CD4^+^ Tregs) with less risk of the immune-related adverse events (irAEs) that currently limit therapy with CAR T cells. Another possible translational application of such knowledge would be in pre-selecting autologous self-antigen-specific CD4^+^ T cells (including Tregs) as the starting material for CAR T cell generation, in order to favour the development of more therapeutically efficacious final CAR T cell product for patient infusion. The use of Tregs as a source material for CAR T cell development could also potentially lead to the exciting prospect of generating “dual programme” CAR T cells, with the option of selecting between effector T cell (anti-tumour) and regulatory T cell (immunosuppressive, to prevent irAEs) functional programmes as appropriate to the clinical status of the patient.

Second, which antigen-presenting cells are responsible for activating tumour-specific CD4^+^ T cells in the tumour microenvironment (Fig. 3b)? Although higher MHC II expression in tumours correlates with better clinical outcome in MHC II^+^ cancers [94–96], MHC II expression across cancer types is highly variable and context-dependent [97–99]. It may be that tumour-infiltrating CD4^+^ T cells recognise antigen on MHC II molecules present on “professional” antigen-presenting cells (myeloid cells of the macrophage or DC lineage and B cells) within the tumour or tumour-draining lymphatics. This could possibly occur in a similar manner to what naturally occurs in secondary lymphoid organs during an infection [100–102]. Furthermore, under certain conditions, non-haemotopoietic cells (e.g. epithelial cells) may also acquire the ability to present antigen on MHC II complexes [103–105]. Understanding these mechanisms of antigen priming and/or re-stimulation within the specific tumour microenvironments would be critical to harness the full potential of CD4^+^ T cells in cancer immunotherapy, and may inform rational combinations with therapies targeting antigen-presenting cells.

Finally, as the fundamental role of anti-tumour CD4^+^ T cell responses becomes increasingly apparent, one under-explored area that should be examined would be the dynamics of the interaction between CD4^+^ T cells and myeloid-derived suppressor cells (MDSCs) within the tumour microenvironment (Fig. 3c). MDSCs are myeloid-lineage cells within the tumour microenvironment that exhibit an immature, tolerogenic phenotype. MDSCs are capable of promoting the differentiation and expansion of regulatory T cell populations within the tumour microenvironment [106–108], possibly by presenting self-antigen on MHC II molecules. Conceptually, this situation is the mirror image of the synergistic positive feedback interactions between effector CD4^+^ T cells and activated DCs presenting non-self-antigens that form the nexus driving immunity. Identifying molecular drivers that disrupt the immunosuppressive interactions between MDSCs [109, 110] and Tregs, possibly by simultaneously converting these cells into activated DCs and effector CD4^+^ T cells, respectively, would be highly informative and beneficial to designing more effective immunotherapeutic strategies.

## Conclusions and perspectives

In this review, we present recent literature showing that CD4^+^ T cells are a critical cornerstone of optimal anti-tumour immunity. Engagement of the CD4^+^ T cell compartment is associated with the generation of an effective anti-tumour response, even when CD4^+^ T cells themselves are not the primary immune cell subtype targeted by therapy. We highlight the emerging consensus that the majority of these tumour-specific CD4^+^ T cells are self-reactive, and juxtapose this against observations from preclinical studies that self-antigen-biased regulatory T cells can themselves mount anti-tumour responses when appropriately conditioned. These recent ongoing developments also present fascinating prospects for the engineering of CD4^+^ T cells for ACT. In conclusion, harnessing the full potential of the immune system for cancer immunotherapy will require a deeper understanding of, and rational targeting of self-reactive CD4^+^ T cells to sustain a durable, robust anti-tumour response towards clinical benefit while minimising autoimmunity.
